# A high-throughput pipeline for the design of real-time PCR signatures

**DOI:** 10.1186/1471-2105-11-340

**Published:** 2010-06-23

**Authors:** Ravi Vijaya Satya, Kamal Kumar, Nela Zavaljevski, Jaques Reifman

**Affiliations:** 1 Biotechnology HPC Software Applications Institute, Telemedicine and Advanced Technology Research Center, U.S. Army Medical Research and Materiel Command, Fort Detrick, MD 21702, USA

## Abstract

**Background:**

Pathogen diagnostic assays based on polymerase chain reaction (PCR) technology provide high sensitivity and specificity. However, the design of these diagnostic assays is computationally intensive, requiring high-throughput methods to identify unique PCR signatures in the presence of an ever increasing availability of sequenced genomes.

**Results:**

We present the Tool for PCR Signature Identification (TOPSI), a high-performance computing pipeline for the design of PCR-based pathogen diagnostic assays. The TOPSI pipeline efficiently designs PCR signatures common to multiple bacterial genomes by obtaining the shared regions through pairwise alignments between the input genomes. TOPSI successfully designed PCR signatures common to 18 *Staphylococcus aureus *genomes in less than 14 hours using 98 cores on a high-performance computing system.

**Conclusions:**

TOPSI is a computationally efficient, fully integrated tool for high-throughput design of PCR signatures common to multiple bacterial genomes. TOPSI is freely available for download at http://www.bhsai.org/downloads/topsi.tar.gz.

## Background

Rapid and accurate identification of pathogens from environmental and clinical samples is essential for effective containment of infectious diseases. Sequence-based identification methods, such as DNA microarrays and polymerase chain reaction (PCR) assays, are effective tools for pathogen diagnostics. Whereas PCR-based assays provide high specificity, microarray-based assays provide high multiplexing capability, accommodating thousands of oligonucleotide probes in a single diagnostic assay. The importance and utility of sequence-based identification methods have further increased in recent times due to advances in DNA sequencing technology that have led to the availability of a large number of pathogen genomes.

A variety of tools have been developed in the past few years to facilitate the design of pathogen-based diagnostic assays [1-10]. Notable among these are the whole-genome based signature design tools KPATH [6], Insignia [3,11], and TOFI [8]. Whereas KPATH designs signatures for PCR-based diagnostic assays and TOFI designs signatures for microarray-based assays, Insignia finds unique sequence segments that can be used to design both PCR and microarray signatures. While, among many features, these tools have the capability to identify common signatures shared by multiple target genomes, each has its own limitations. For example, KPATH computes consensus regions among the target genomes from their multiple alignments. However, multiple alignment of whole bacterial genomes is computationally intensive and it is not practical when a large number (> 20) of genomes is to be analyzed. Conversely, TOFI and Insignia build consensus regions among multiple genomes through pairwise alignments between the target genomes. Insignia server reports only the unique segments in the target genomes and provides an option for users to run the Primer3 [12] PCR signature design software on these unique segments. Manual manipulation is necessary to extract the PCR signatures from Primer3 outputs and to perform further specificity analysis on the extracted signatures. Further manual manipulation is necessary when the unique segments reported by Insignia are not long enough to accommodate a complete PCR signature, in which case PCR signature components have to be designed individually from smaller unique segments close to each other, and the individual components have to be manually assembled to form valid PCR signatures. Insignia is extremely fast, as it precomputes the matches between all pairs of sequences. However, this advantage in speed comes with a limitation; the user is restricted to genomic sequences that are part of the Insignia database, and does not possess an option to use other sequences as targets or non-targets. The TOFI pipeline is free of most of the limitations described above, but can only design signatures for microarray-based diagnostic assays.

In this paper, we describe the Tool for PCR Signature Identification (TOPSI), which extends the TOFI framework [7-9] to design signatures for real-time PCR-based diagnostic assays. Like Insignia, TOPSI uses pairwise alignments to identify sequences that are common to multiple genomes, and compares these sequences with non-target genomes to identify unique segments suitable for designing signatures. However, TOPSI goes beyond the identification of unique segments, and incorporates modules to design PCR signatures from the unique segments and perform extensive specificity analysis on the designed signatures. Being fully integrated and automated, TOPSI takes a set of input target sequences and provides a list of PCR signatures common to all input targets without the need for manual intervention in any of the intermediate steps. Unlike existing software systems for real-time PCR signature design, TOPSI is the only one that is: freely available, high-throughput, and fully integrated. The following are some of the unique features of TOPSI:

• *Highly scalable*: TOPSI is very efficient and scalable, as a result of using pairwise alignments as opposed to multiple genome alignments.

• *Fully integrated and automated*: Complete PCR signature design and comprehensive specificity analysis are an integral part of TOPSI. PCR primers and probes are directly provided to the user, without the necessity for any manual manipulation.

• *Freely available*: TOPSI is freely available for download and installation, giving users complete control over the selection of non-target databases and ensuring user confidentiality of the applications.

The TOPSI pipeline has been primarily designed to work with a large number of bacterial genomes. Designing common signatures for multiple viral genomes offers a different set of computational challenges. Although viral genomes are much smaller in size, signature design is complicated by the high variability within such genomes and the consequent lack of conserved regions suitable for signature design. As a result, the current TOPSI framework might not be successful in designing signatures common to a large number of viral genomes.

## Implementation

**Figure 1:**
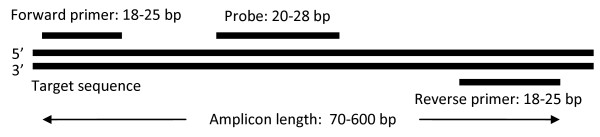
**Components of a real-time PCR signature**. A real-time PCR signature consists of a forward primer, a probe, and a reverse primer. The lengths of these three components, the distances between them, and the total amplicon length can vary greatly depending on the real-time PCR technology. The values shown in the figure are typical for the TaqMan^® ^real-time PCR assays.

A real-time PCR signature consists of two primers and a probe, as shown in Figure 1. The two primers facilitate PCR amplification of the target sequence, whereas the probe serves to report the amplified product. The lengths and thermodynamic properties of the three components vary across different real-time PCR technologies. Although the default parameters in the TOPSI pipeline have been selected to be compatible with the TaqMan^® ^real-time PCR system, TOPSI allows the user to customize all Primer3 input parameters, thereby enabling the user to design signatures for any PCR platform for which Primer3 can design signatures.

### Overview of the pipeline

**Figure 2:**
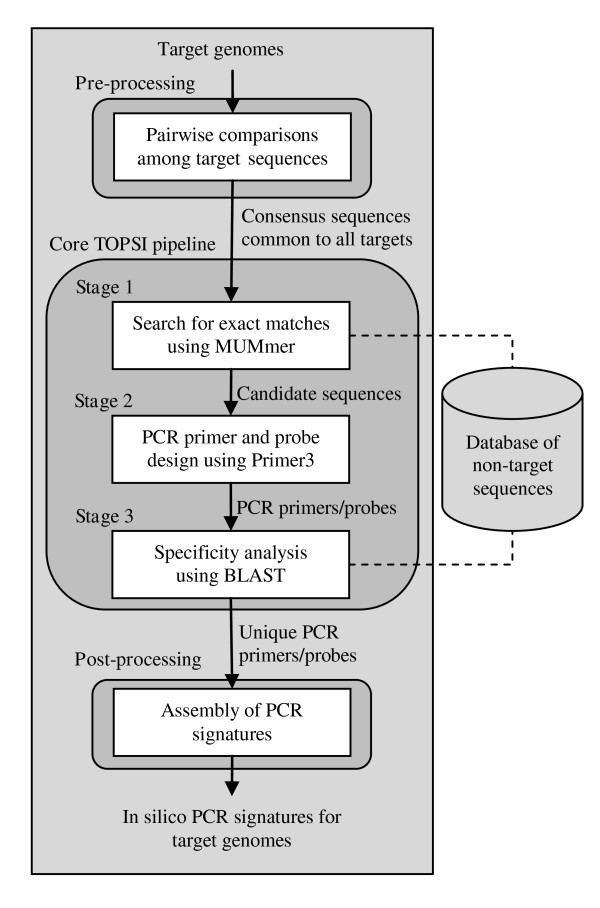
**Overview of the TOPSI pipeline**. The pre-processing stage of TOPSI obtains consensus sequence segments that are common to all input genomes. The actual signature design process, including comparison with non-target genomes, is performed in the three stages of the core TOPSI pipeline. The post-processing module assembles individual unique primers and probes into PCR signatures.

In the following, we briefly describe the TOPSI pipeline for real-time PCR signature design. The TOPSI pipeline consists of a pre-processing step, a post-processing step, and three main stages as shown in Figure 2. Similar to the previously developed TOFI pipeline [7-9], the different stages are designed so that large portions of the target genomes are eliminated in the less-expensive initial stages, and the computationally expensive specificity analysis is performed over smaller regions of the target genomes in the third stage of TOPSI. All three stages of the core TOPSI pipeline are executed in parallel on multiple processors.

The input genomes are first compared with each other using the suffix-tree-based MUMmer [13] program in the pre-processing stage of TOPSI. Starting with an arbitrary pair of input genomes, pairwise local alignment is performed between the two genomes, and a list of conserved sequence segments that are shared between the two genomes is constructed from the pairwise alignment. This list of conserved sequence segments is then sequentially compared with each of the remaining input genomes and continually updated so as to contain only those sequence segments that are shared among all the input genomes. Designing PCR signatures from these conserved sequence segments ensures that each of the input genomes is amplified by the designed signatures.

The first stage of the core TOPSI pipeline uses the MUMmer program to perform pairwise comparison of the conserved target sequences with each non-target genome. This step eliminates any segments in the input sequences that have exact matches longer than a user-specified length with any of the non-target genomes in a comprehensive sequence database, such as the *nt *database provided by the National Center for Biotechnology Information (NCBI). The surviving segments, referred to as the candidate sequences, are then passed on to the second stage of the pipeline.

In the second stage, TOPSI uses the open source Primer3 [12] software to identify primers and probes with the desired thermodynamic properties from the candidate sequences. At this stage of the pipeline, forward primers, reverse primers, and probes are designed independently, without taking into consideration the distance constraints between the primers and probes. This approach ensures that all unique primers and probes are reported, which can later be used to design PCR signatures in which only one, two or all three of the components are unique.

The third stage of TOPSI performs specificity analysis by performing BLAST [14] alignments of each primer and probe with any comprehensive sequence database provided by the user, such as the *nt *database. The BLAST alignments are performed in parallel on multiple processors using the blastn program of the parallel BLAST implementation mpiBLAST [15]. Based on the BLAST alignments, primers and probes with significant alignments to non-target genomes are eliminated. The output of this stage consists of individual primers and probes that are unique to the target genome(s).

In the final post-processing step, the individual unique PCR primers and probes are assembled into PCR signatures by taking distance constraints into consideration. First, PCR signatures with all three unique components are identified. Further, PCR signatures with one or two unique components are also designed by taking each unique PCR primer or probe and designing non-unique components by running Primer3 on the conserved target sequence segments on either side of the unique components. These PCR signatures with one or two unique components are useful when there are very few or no PCR signatures with all three unique components.

### Criteria for specificity analysis

TOPSI uses a combination of multiple criteria for performing specificity analysis, similar to TOFI [9]. These criteria include the maximum percentage identity, the longest stretch of contiguous/near-contiguous matches, and the minimum number of mismatches with a non-target sequence. All these different criteria are evaluated based on the BLAST alignments obtained with non-target sequences. Whereas thresholds on the maximum percentage identity and the longest stretch of contiguous or near-contiguous matches are useful in evaluating the specificity of longer sequences, thresholds for the minimum number of mismatches with non-target sequences are useful in evaluating the specificity of short primer or probe sequences. Combining these criteria ensures that probes and primers of all different lengths are specific to the target genomes.

### Signature ranking

To enable the user to select a subset of the *in silico *designed PCR signatures, TOPSI assigns two different scores to each PCR signature. The first score, called uniqueness penalty, is a measure of the specificity of the PCR signature. The uniqueness penalty of each component of the PCR signature is calculated based on the best non-target match and the length of the longest contiguous match with a non-target sequence. A primer or probe with overall identity or the longest contiguous match exceeding pre-specified thresholds supplied by the user is assigned a penalty score of 1. Conversely, a primer or probe with no significant matches with non-target sequences is assigned a uniqueness penalty of zero. The uniqueness penalty for a PCR signature is computed as the sum of the uniqueness penalties of the individual components. The second score computed by TOPSI is the sum of the penalty scores reported by Primer3 for each of the three components, and is a measure of how close the thermodynamic properties of these components are to the optimal parameters selected by the user. The user can select a subset of the PCR signatures by ranking the probes based on any one of these scores, or by using a combined score calculated by assigning customized weights to each of the two scores.

## Results

In this section, we report signatures designed by TOPSI and compare them with those designed by other software systems as well as some experimentally verified signatures. We also discuss potential limitations of TOPSI.

### Performance of the TOPSI pipeline

To evaluate the performance of TOPSI on a large set of genomes, we ran TOPSI for a set of 18 *Staphylococcus aureus *genomes using 98 cores of a Linux cluster with distributed memory. Table 1 shows the details of the 18 *S. aureus *genomes used. The combined size of these 18 genomes was greater than 50 Mbp. We used the NCBI *nt *database (size ~30 Gbp) as the non-target sequence database. The pre-processing step was very efficient, and obtained consensus sequence segments shared among all the 18 genomes in less than 10 minutes. Stage 1 and Stage 2 of the core TOPSI pipeline were also very fast, and finished within 50 and 15 minutes, respectively. As expected, Stage 3 was the most computationally intensive part of TOPSI. For this instance, Stage 3 of the TOPSI pipeline took nearly 12 hours. The total execution time of the pipeline was approximately 13.5 hours, illustrating that TOPSI is extremely efficient in designing PCR signatures for a large number of genomes.

**Table 1:** List of *S. aureus *genomes used for comparing TOPSI and KPATH

Strain	Source	NCBI Taxon ID
*S. aureus *subsp. aureus Mu50	Refseq:NC_002758	158878
*S. aureus *subsp. aureus COL	Refseq:NC_002951	93062
*S. aureus *subsp. aureus JH1	Refseq:NC_009632	359787
*S. aureus *subsp. aureus JH9	Refseq:NC_009487	359786
*S. aureus *subsp. aureus MRSA252	Refseq:NC_002952	282458
*S. aureus *subsp. aureus MSSA476	Refseq:NC_002953	282459
*S. aureus *subsp. aureus Mu3	Refseq:NC_009782	418127
*S. aureus *subsp. aureus MW2	Refseq:NC_003923	196620
*S. aureus *subsp. aureus N315	Refseq:NC_002745	158879
*S. aureus *subsp. aureus NCTC 8325	Refseq:NC_007795	93061
*S. aureus *subsp. aureus str. Newman	Refseq:NC_009641	426430
*S. aureus *RF122	Refseq:NC_007622	273036
*S. aureus *subsp. aureus USA300 FPR3757	Refseq:NC_007793	451515
*S. aureus *subsp. aureus USA300 TCH1516	Refseq:NC_010079	451156
*S. aureus *EMRSA15 draft sequence	Sanger Institute	N/A
*S. aureus *0582 draft sequence	Sanger Institute	N/A
*S. aureus *subsp. aureus str. JKD6008	Refseq:NZ_ABRZ00000000	546342
*S. aureus *subsp. aureus str. JKD6009	Refseq:NZ_ABSA00000000	546343

### Comparison with other software systems

To evaluate the PCR signatures designed by TOPSI, we compared them with signatures obtained from KPATH [6]. The developers of KPATH provided us with 1236 KPATH signatures for the 18 *S. aureus *genomes listed in Table 1. We obtained 719 PCR signatures by running TOPSI with the strict specificity thresholds listed in Table 2. By using the relaxed thresholds listed in Table 2 to effectively eliminate some specificity criteria, we obtained 2430 signatures. This indicates that the number of signatures reported by TOPSI is comparable to that obtained by KPATH. In total, there are 830 TOPSI signatures that overlap with the KPATH signatures. It is important to note that a direct mapping of TOPSI to KPATH signatures is not possible because very few signatures will be exactly the same in the two software systems. This is due to small differences in the primer/probe design criteria and in the input processing that lead to the selection of a different set of PCR primers or probes in the same region.

**Table 2:** Specificity thresholds used in TOPSI runs for *S. aureus*

Specificity parameter	Strict threshold	Relaxed threshold
M0 - longest stretch of contiguous matches with a non-target that has no mismatches	18	18
M1 - longest stretch of contiguous matches with a non-target that has at most one mismatch	20	40
M2 - longest stretch of contiguous matches with a non-target that has at most two mismatches	22	40
M3 - longest stretch of contiguous matches with a non-target that has at most three mismatches	24	40
Maximum overall identity with a non-target sequence	90%	90%

**Figure 3:**
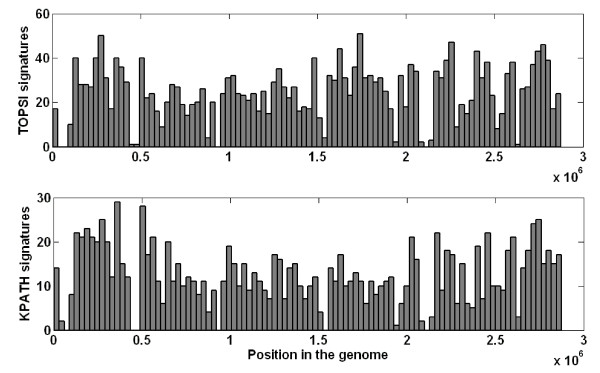
**Distribution of TOPSI and KPATH signatures in the *S. aureus *Mu50 genome**. The distributions shown here are for the 1236 KPATH signatures and the 2430 TOPSI signatures obtained using relaxed thresholds. Both TOPSI and KPATH signatures are distributed throughout the *S. aureus *genome. The regions in which there are very few or no TOPSI signatures also have very few or no KPATH signatures, indicating that these regions are in fact not suitable for PCR signature design.

Figure 3 shows the distribution of TOPSI and KPATH signatures in the *S. aureus *Mu50 genome. It can be seen that both the TOPSI and the KPATH signatures are distributed throughout the genome. In the regions where TOPSI does not report any signature, KPATH also does not report any signature, indicating that these regions are not suitable for designing unique PCR signatures. These results provide semi-quantitative validation that the number of signatures reported by TOPSI is similar to that reported by KPATH, and that the TOPSI signatures are distributed throughout the genome without the conspicuous omission of any region for which PCR signatures have been reported by KPATH.

We attempted to obtain Insignia signatures using a total of 21 *S. aureus *genomes that were accessible through the Insignia Web server as of 4 June 2010, selecting the option to include NCBI RefSeq among non-targets and using a signature word length of 18 to match the corresponding parameter in TOPSI. Insignia produced a set of 68,879 signature chains (i.e., candidate regions for signature design) in less than a minute. As these signatures were too many for the Insignia Web server to run Primer3 or BLAST, we used the length filter to obtain 1,702 signature chains with length ≥ 28 bp. However, running Primer3 using the default parameters did not produce any PCR primers or probes, as the signature chains were not long enough (each was ≤ 51 bp) to accommodate all three PCR components. Individual PCR components could be designed independently, but Insignia does not provide any module to assemble adjacent PCR components into complete PCR signatures. Given that there were thousands of signature candidates, it was impractical to assemble the PCR components manually for the entire genome. To estimate the time necessary for specificity analysis, we used the BLAST search option in Insignia to submit 400 signature chains to the NCBI BLAST Web server, which took 1 hr and 26 minutes to produce the results. Assuming that the average time per query remains the same, it would take ~10 days to perform the BLAST analysis on the original 68,879 signature chains returned by Insignia. These results suggest that although Insignia might be extremely useful and convenient for designing a few signatures from selected regions of the target genome, unlike TOPSI, it is not ideal for high-throughput, whole-genome signature design on bacterial genomes that might result in thousands of signature candidates.

### Comparison with experimentally verified signatures

To compare TOPSI signatures with experimentally verified signatures, we selected the extremely difficult case of PCR signatures that are unique to *Burkholderia mallei *with respect to *Burkholderia pseudomallei*. *B. mallei *and *B. pseudomallei *are closely related pathogens that cause different diseases, glanders and melioidosis, respectively [16]. *B. mallei *is believed to have been clonally evolved from *B. pseudomallei *[17], with a significantly reduced genome due to the loss of genes. Due to the similarity of *B. mallei *with respect to *B. pseudomallei*, a literature search revealed only one PCR signature that was reported to be unique to *B. mallei *[16]. However, both primers in this signature (5'-TTCGATCGATTCCTGCTATC-3' and 5'-GCGTTAAACGCCGTACTTTC-3') have exact matches with some newly sequenced *B. pseudomallei *strains. The Web-based tool Primer-BLAST http://www.ncbi.nlm.nih.gov/tools/primer-blast/index.cgi predicts that these primers will amplify *B. pseudomallei *strains 33, 172, 491, and 668. Hence, this PCR signature can no longer be considered to be unique to *B. mallei*.

Another set of 10 experimentally validated *B. mallei *specific PCR signatures were available from the Center for Bioinformatics and Computational Biology at the University of Maryland http://insignia.cbcb.umd.edu/pdf/burkholderia.pdf. These real-time PCR signatures were designed through their Insignia [3] system. However, BLAST comparisons of the primers and probes comprising these signatures with the NCBI whole-genome shotgun sequence (WGS) database in October 2009 revealed that five of these signatures did not meet our design criteria. Reasons for eliminating these signatures are listed in Additional file 1. The remaining five PCR signatures were still unique to *B. mallei *sequences.

**Figure 4:**
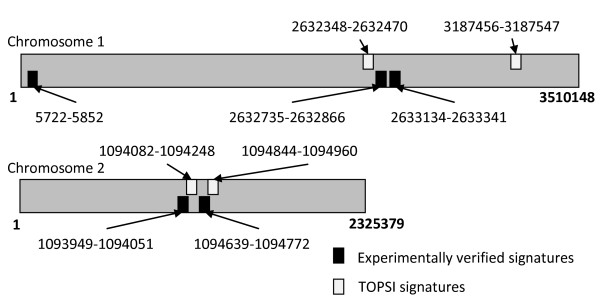
**Comparison of TOPSI signatures with experimentally verified signatures for *B. mallei***. The four TOPSI signatures and five experimentally verified signatures provided by the University of Maryland are mapped to the *B. mallei *ATCC 23344 genome.

We ran TOPSI with 11 *B. mallei *genomes shown as targets, using a non-target database which included the NCBI *nt *database and 23 *B. pseudomallei *genomes. Table 3 shows the 11 *B. mallei *genomes used. With these inputs, TOPSI designed 11 real-time PCR signatures in which all three components were unique to and present in all the 11 *B. mallei *genomes. However, seven out of these 11 signatures were eliminated when compared against the WGS database, due to matches with draft non-target genomes. Table 4 shows the remaining four TOPSI signatures. Figure 4 shows the relative positions of these four TOPSI signatures and the five experimentally verified signatures from the University of Maryland (described above). It can be seen that three of the four TOPSI signatures are within 300 bp of one of the five experimentally verified signatures. This indicates that TOPSI was successful in identifying the experimentally verified unique regions of *B. mallei*. One TOPSI signature and one experimentally verified signature do not have any counterparts in their immediate neighborhoods.

**Table 3:** List of 11 *B. mallei *genomes used for designing common signatures

Strain	Source	NCBI Taxon ID
*B. mallei *ATCC 23344	Refseq:NC_006348, Refseq:NC_006349	243160
*B. mallei *NCTC 10229	Refseq:NC_008836, Refseq:NC_008835	412022
*B. mallei *NCTC 10247	Refseq:NC_009080, Refseq:NC_009079	320389
*B. mallei *SAVP1	Refseq:NC_008785, Refseq:NC_008784	320288
*B. mallei *PRL-20	JCVI	436115
*B. mallei *PRL7	JCVI	536228
*B. mallei *2002721280	JCVI	370895
*B. mallei *ATCC 10399	JCVI	412021
*B. mallei *FMH	JCVI	334802
*B. mallei *GB8 horse 4	JCVI	320390
*B. mallei *JHU	JCVI	334803

**Table 4:** TOPSI signatures for *B. mallei*.

Chr.	Forward primer (position)	Probe (position)	Reverse primer (position)	Amplicon length
1	ACTGCTGTACCGCGCTCTTT (2632348)	ATTCGCAGCACCCATTACAACCGTTG (2632376)	GCTGAAGAGTGGCTGCAATG (2632449)	121
1	GGTCTACAGCTCCGCGAATT (3187456)	CTCAAACCGTTAGGCGACTCAAGGTGC (3187488)	ATGGCATGGTGCTGTGAAAC (3187528)	92
2	CGAGCCATCGACCTCATG (1094082)	ACATGTCGAAGCATTTTTCGCCGC (1094188)	TCGGCGATCGATGGTCTAG (1094228)	165
2	CTCCCACGCCGACTGATAC (1094844)	AAGCTGTTGATCGTGCAACACCAGCAC (1094883)	CGGTGAAACCTGGATACTGGA (1094938)	115

The positions and amplicon lengths are with reference to the *B. mallei *ATCC 23344 genome.

### Potential limitations of TOPSI

One potential limitation of TOPSI, and of other similar high-throughput signature design software systems, is their difficulty in designing signatures for viral genomes, as described by Philippy *et al*.[3]. Because of their small genomes and high variability, it may not be possible to find conserved segments to design signatures from. We tested TOPSI with two viral agents, Variola major and human adenovirus. TOPSI identified six *in silico *PCR signatures (with at least one unique segment) common to 40 Variola major genomes, using three Variola minor genomes as non-targets, based on the classification provided by Esposito *et al*. [18]. In contrast, TOPSI could not identify any common signatures for 16 human adenovirus genomes consisting of subgroups A, B, C, D, E and F. However, TOPSI could design unique PCR signatures common to two genomes of human adenovirus subgroup D. Our experience based on this limited testing with viral genomes indicates that it might be possible for TOPSI to design common signatures for large DNA viruses. However, TOPSI might not be able to design common signatures for short RNA viruses, in which case methods specifically designed for viral genomes, such as the one described by Duitama *et al*. [19], need to be incorporated.

Another issue of concern is the effect of draft or incomplete genomes on signature design. In the current TOPSI framework, PCR signatures are designed from genomic regions that are conserved among all the input genomes. This might potentially lead to a situation in which signatures common to a large number of input genomes are eliminated because of a single low-quality or incomplete genome sequence. One possible solution for this problem is to apply a lower threshold for consensus, so that signatures can be designed from regions that are conserved among a large percentage of the input genomes. This approach is compatible with the current TOPSI framework and could be incorporated into the system. However, this solution would lead to signatures that do not identify some of the target genomes. Therefore, it should be used only when signatures common to all targets cannot be identified. Another solution using the current TOPSI framework is to design signatures based solely on finished genomes as a first step, and subsequently filter the obtained signatures by applying a threshold on the percentage of draft (or incomplete) genomes that are identified by each signature. Alternatively, if sequence quality scores are available, taking them into consideration while evaluating the consensus regions might also lead to identifying signatures that might otherwise be eliminated.

## Conclusions

The TOPSI pipeline is efficient in designing real-time PCR signatures that are common to multiple strains of a bacterial pathogen, and are also unique to the pathogen with respect to all other sequenced non-target genomes. Comparison with PCR signatures designed using a well-established software system shows that the TOPSI signatures are similar to those designed by the other software, and comparison with experimentally verified signatures shows that TOPSI is able to report signatures from unique regions of the pathogen genome. Being the only freely available, high-throughput, and fully integrated solution for the design of real-time PCR signatures, TOPSI provides a valuable contribution to the development of pathogen diagnostic assays.

## Availability and requirements

• **Project name: **TOPSI

• **Project home page: **http://www.bhsai.org/downloads/topsi.tar.gz

• **Operating systems: **Linux

• **Programming language: **Perl

• **Other requirements: **mpiBLAST 1.4.0 or later, MUMmer 3.19 or later, Primer3 1.1.4 or later, BioPerl, and a Linux cluster with PBS queuing system

TOPSI is also operational as a Web server at a U.S. Department of Defense (DoD) high-performance computing center. Sponsorship for access to these resources may be requested by contacting the corresponding author.

## Authors' contributions

RVS implemented the various modules in TOPSI, and KK developed the user interface. NZ compared the TOPSI signatures with the experimentally verified signatures. JR conceived the project and provided overall project guidance. RVS, NZ, and JR were the main writers of the manuscript. All authors read and approved the final manuscript.

## Supplementary Material

Additional file 1 **Reasons for eliminating some experimentally verified *B. mallei *signatures**. Detailed explanation for eliminating five out of the ten experimentally verified *B. mallei *signatures provided by the University of Maryland http://insignia.cbcb.umd.edu/pdf/burkholderia.pdf. Click here for file
