# Supplementary material for: A high-throughput pipeline for the design of real-time PCR signatures
Source: BMC Bioinformatics. 2010 Jun 23;11:340. doi: 10.1186/1471-2105-11-340 (PMC2905370; doi:10.1186/1471-2105-11-340)
Supplement: Additional file 1 — Reasons for eliminating some experimentally verified B. mallei signatures. Detailed explanation for eliminating five out of the ten experimentally verified B. mallei signatures provided by the University of Maryland http://insignia.cbcb.umd.edu/pdf/burkholderia.pdf. [file 1471-2105-11-340-S1.DOCX]

**Additional file1 – Reasons for eliminating five out of the ten experimentally verified *B. mallei* signatures provided by University of Maryland (http://insignia.cbcb.umd.edu/pdf/burkholderia.pdf)**

| Sig. # | Region | Forward primer  Probe  Reverse primer | Reasons for elimination |
| --- | --- | --- | --- |
| 92 | Chr2,  834580-834684 | GTCGTGCAATCGACGTTC  TACAGCCCATTCGACAGGTCCGTC  GGTCGGACATCATGCTGAG | All three components have exact matches with the following *B. pseudomallei* genomes:  576 BUC.Contig175 [ACCE01000014.1](http://www.ncbi.nlm.nih.gov/entrez/query.fcgi?cmd=Retrieve&db=Nucleotide&list_uids=217391551&dopt=GenBank&RID=VRE05V0H01S&log$=nucltop&blast_rank=2)  MSHR346 [NZ_ACOJ01000001.1](http://www.ncbi.nlm.nih.gov/entrez/query.fcgi?cmd=Retrieve&db=Nucleotide&list_uids=237507289&dopt=GenBank&RID=VRE05V0H01S&log$=nucltop&blast_rank=1)  BCC215 [ABBR01000791.1](http://www.ncbi.nlm.nih.gov/entrez/query.fcgi?cmd=Retrieve&db=Nucleotide&list_uids=148651025&dopt=GenBank&RID=VRE05V0H01S&log$=nucltop&blast_rank=3)  NCTC 13177 [ABBQ01000832.1](http://www.ncbi.nlm.nih.gov/entrez/query.fcgi?cmd=Retrieve&db=Nucleotide&list_uids=148649953&dopt=GenBank&RID=VRE05V0H01S&log$=nucltop&blast_rank=4) |
| 93 | Chr1,  787807-787913 | AAAGCAGCAGACGATCCATT  CTTATGTGTGACTGCGGCTGGCAC  ACTCGAAGACGTAAGCCCG | All three components were found to be insensitive to one of the targets, i.e., they do not match *B. mallei* SAVP1 ([NC_008784](http://www.ncbi.nlm.nih.gov/sites/entrez?Db=genome&Cmd=ShowDetailView&TermToSearch=20275), [NC_008785](http://www.ncbi.nlm.nih.gov/sites/entrez?Db=genome&Cmd=ShowDetailView&TermToSearch=20276)) |
| 94 | Chr1,  2633324-2633438 | TAGGGTAGGTTCCGAGGGTT  GTTTGATGCATGTCGGGCCTCCTA  GAGCGGGTCTTACGTTTTCA | Our design criteria require the probe to be at least one bp away from each of the primers, and at most 40 bp away from at least one of the primers in all the target genomes. In case of this signature, we found that the probe and the reverse primer are adjacent to each other (with no gap) in *B. mallei* ATCC 23344 [CP000010.1](http://www.ncbi.nlm.nih.gov/entrez/query.fcgi?cmd=Retrieve&db=Nucleotide&list_uids=52426793&dopt=GenBank&RID=VT2JSYW5012&log$=nucltop&blast_rank=4).  In addition, the forward primer matches *Drosophila erecta* [AAPQ01006920.1](http://www.ncbi.nlm.nih.gov/entrez/query.fcgi?cmd=Retrieve&db=Nucleotide&list_uids=91889809&dopt=GenBank&RID=WT4ATY5N016&log$=nucltop&blast_rank=7) and the probe matches *B. thailandensis* [ABBM01000536.1](http://www.ncbi.nlm.nih.gov/entrez/query.fcgi?cmd=Retrieve&db=Nucleotide&list_uids=148644031&dopt=GenBank&RID=WT4BCY8V016&log$=nucltop&blast_rank=7). |
| 98 | Chr1,  2632337-2632488 | ACCCATTACAACCGTTGCTG  GCCCCAGTCCTCCCGATGCC  GCTGAAGAGTGGCTGCAAT | Same reason as above. No gap between the probe and reverse primer in *B. mallei* ATCC 23344 [CP000010.1](http://www.ncbi.nlm.nih.gov/entrez/query.fcgi?cmd=Retrieve&db=Nucleotide&list_uids=52426793&dopt=GenBank&RID=VT2JSYW5012&log$=nucltop&blast_rank=4).  In addition , both the primers match *B. thailandensis* [ABBM01000536.1](http://www.ncbi.nlm.nih.gov/entrez/query.fcgi?cmd=Retrieve&db=Nucleotide&list_uids=148644031&dopt=GenBank&RID=WT4BCY8V016&log$=nucltop&blast_rank=7), and the probe matches *Echinops telfairi* [AAIY01462640.1](http://www.ncbi.nlm.nih.gov/entrez/query.fcgi?cmd=Retrieve&db=Nucleotide&list_uids=72954986&dopt=GenBank&RID=WT51744U012&log$=nucltop&blast_rank=7). |
| 99 | Chr2,  909144-909329 | CTCTATCGAATTTCGGCTCG  AGAATCTCGCCACACTCTCGCCAC  TTACGATTTCGCCTTAACCG | All three components have exact matches with the *B. pseudomallei* 112 [ABBP01000271.1](http://www.ncbi.nlm.nih.gov/entrez/query.fcgi?cmd=Retrieve&db=Nucleotide&list_uids=148649436&dopt=GenBank&RID=VREV4KV001N&log$=nucltop&blast_rank=1). |
